# Environmental Triggering of Type 1 Diabetes Autoimmunity

**DOI:** 10.3389/fendo.2022.933965

**Published:** 2022-07-22

**Authors:** Pamela Houeiss, Sandrine Luce, Christian Boitard

**Affiliations:** ^1^ Laboratory Immunology of Diabetes, Department EMD, Cochin Institute, INSERMU1016, Paris, France; ^2^ Medical Faculty, Paris University, Paris, France

**Keywords:** type 1 diabetes, genetics, pathogenesis, environmental factors, adaptive immunity, innate immunity, beta cells

## Abstract

Type 1 diabetes (T1D) is a chronic autoimmune disease in which pancreatic islet β cells are destroyed by immune cells, ultimately leading to overt diabetes. The progressive increase in T1D incidence over the years points to the role of environmental factors in triggering or accelerating the disease process which develops on a highly multigenic susceptibility background. Evidence that environmental factors induce T1D has mostly been obtained in animal models. In the human, associations between viruses, dietary habits or changes in the microbiota and the development of islet cell autoantibodies or overt diabetes have been reported. So far, prediction of T1D development is mostly based on autoantibody detection. Future work should focus on identifying a causality between the different environmental risk factors and T1D development to improve prediction scores. This should allow developing preventive strategies to limit the T1D burden in the future.

## Introduction

Type 1 diabetes (T1D) is an autoimmune disease in which immune cells infiltrate the islets of Langerhans of the pancreas, ultimately destroy insulin secreting β cells and later lead to overt T1D. The kinetics of islet β cell destruction along the development of insulitis remains an open issue and may differ between patients (1). T1D is a multifactorial disease. The progressive increase in T1D incidence in all countries in which it has been evaluated over the last 60 years points to the role of environmental factors in triggering or accelerating the disease process (2). Immune mechanisms involved in the autoimmune attack against β cells have been mostly defined in T1D models in the rat and in the mouse (3). Although they may apply to the human, human T1D is likely to be a heterogeneous disease in which all patients do not follow the same disease process. It is commonly proposed that an environmental factor triggers an immune response in islet beta cells mediated by T lymphocytes in genetically susceptible individuals (4). However, many environmental factors have been identified regardless of whether they trigger the initial seroconversion to anti-islet cell antibodies or accelerate the disease process in autoantibody-positive individuals (5–9). The first events associated with the disease process is an increased expression of class I major histocompatibility genes and the expression of interferon α and homing genes (10). In an era where the pathogenesis of the disease is not fully elucidated and a cure is still lacking, the identification of triggering factors is crucial for prevention. This review article will focus on the possible risk factors of T1D. It will describe hypothesis behind T1D triggering in the human.

T1D is, in its most common form, a chronic disease. It progresses over 3 stages (11). The first is defined by the detection of autoantibodies in the absence of hyperglycemia, i.e. prediabetes. The second starts when hyperglycemia develops and the third is when overt diabetes develops and clinical symptoms reveal the diabetes process. Whether the loss of β-cells follows a progressively decreasing slope or a relapsing remitting-like pattern remains unknown (12). In the human, autoantibodies are the biological marker for autoimmune diabetes, although they do not play a significant role in β-cell destruction. Anti-insulin (IAA), anti-glutamic acid decarboxylase (anti-GAD), anti-islet antigen 2 (anti-IA2), and anti-zinc transporter-8 (ZnT8) autoantibodies are detected in up to 100% of children developing diabetes before 5 years of age and with a lower prevalence in individuals who may only carry some of these autoantibodies (13). They are predictive of diabetes in first degree relatives of patients with T1D or newborns from a T1D parent. They are detected months or years before the development of the disease depending on the age at onset. Having 2 or more antibodies before the age of 3 is associated with a risk of 75% to develop T1D at 10 years. Having all 4 antibodies implicate that 100% of patients will develop T1D upon a 20 year follow up (13).

## Pathogenesis

**Figure 1:**
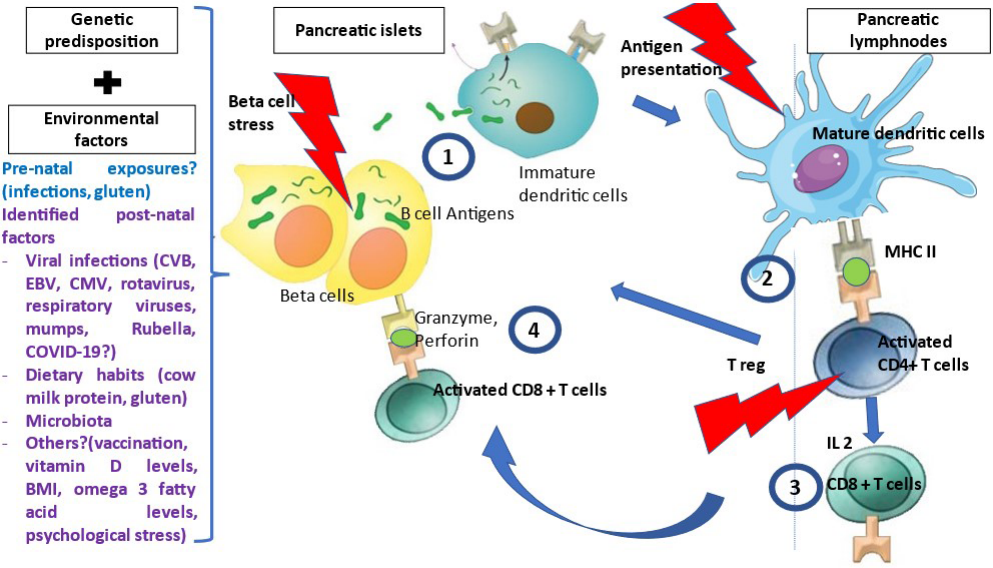
Pathophysiology of T1D. Many environmental factors were associated with T1D development. The initial mechanism that triggers autoimmunity is unknown. In genetically susceptible patients, in the presence of environmental factors, β cells are stressed and release β cell antigens. **1-** β cell autoantigens are processed by APCs and presented by HLA class II MHC molecules to the naïve CD4+T-cells in the peripheral LN. **2-** CD4+ T-cells are activated and polarized towards a TH1 phenotype releasing cytokines, among which IL 2, INF gamma and IL-4 at the expense of regulatory T cells (T reg). **3**- This will activate and generate auto-reactive CD8+ T-cells, which recognized autoantigen peptides presented by HLA class I MHC molecules, migrate to the islets and liberate cytotoxic granzyme and perforin to destroy β cells.

T1D is a T-cell mediated disease that is likely driven by events occurring at the islet level, i.e. danger signals (**Figure 1**) (14). Therefore, autoantigens are processed and presented by dendritic cells issued from the islets and that migrate to satellite lymph nodes where they drive the clonal expansion of autoantigen-specific CD4^+^ T-cells and secondarily CD8^+^ T-cells and B-cells. Along their activation, T and B-cells modify their expression of homing genes and migrate back to the islets where they expand and mediate β-cell destruction (15). Other potential players are currently being investigated such as FasL-expressing B cells and dual expresser (DE) cells (16). Autoantigens recognized by T-cells include the four main autoantigens corresponding to those targeted by autoantibodies and chromogranin, islet-glucose-6-phosphatase catalytic subunit-related protein (IGRP), islet amyloid polypeptide and glial fibrillary acidic protein (17–19). Anti-insulin is the first antibody to be detected in patients at risk for T1D. Preproinsulin (PPI) epitopes that are presented by different HLA class I molecules to CD8^+^ T-cells and by HLA class II molecules to CD4^+^ T-cells have been characterized (17). They span along the whole sequence of proinsulin, the insulin prohormone precursor and within the 24 amino acid signal peptide sequence of the protein (20). The role of insulin as a major autoantigen has been demonstrated in the Non-Obese diabetic (NOD) mouse, as the knockout of the *insulin 1* gene prevents the development of type 1 diabetes while the knockout of the *insulin 2* gene accelerates the disease process (21). PPI-specific CD8^+^ and CD4^+^ T-cell are detected in the peripheral blood and in islets in the mouse and in the human (22–25). A missing piece of the puzzle is the triggering event that leads to increased class I expression, expression of interferon α and homing genes and dendritic cell activation and migration.

## Genetic Risk Factors

If one except rare monogenic forms (26), T1D is a multifactorial disease that involve 60 gene variants that have been associated to T1D along with multiple environmental factors (27). Investigating the role of these factors in prediabetes and diabetes is complex and requires longitudinal studies as well as separate analysis for the two conditions. Monozygotic twins show a 30 to 50% concordance for T1D, pointing again to the role of environmental factors. Individuals with an affected first degree relative have 15 times higher lifetime risk of developing T1D as compared with the general population (5% v/s 0.3%).%) (28).

GWAS studies identified over 60 genes associated with T1D susceptibility (29). These genes operate both in pancreatic β cells and innate and adaptive immune systems (30). Variations in genes involved in central or peripheral tolerance can explain the risk of immune tolerance failure in susceptible individuals. The bioinformatic approach gave us a new way to identify culprit genes by focusing on functional aspects (31). Fine mapping data identified T1D associated loci in lymphocytes’ selection, activation, and regulation. Genes that confer the highest susceptibility are class II HLA genes, followed by *INS*, *CTLA4* and *PTPN22* genes (32, 33). Different genes are associated with different stages of T1D, and some genes are likely to influence disease progression rather than their initiation.

HLA genes contribute to 40-50% of the T1D lifetime risk (34). HLA class II molecules are involved in the initiation of T1D and HLA class I molecules are more likely contributing to disease progression. By activating CD4^+^ T-cells, HLA class II-peptide complexes initiate the autoimmune response. Among class II genes, HLA-DQ alleles carry the highest susceptibility. Susceptibility is attributed to the presence of a non-aspartic amino acid in position B57 in the HLA-DQB1 pocket 9 and in positions 13 and 71 in the HLA-DRB1 chain (35). An odds ratio (OR) of 10 is associated with DR4-DQ8 (DQA1*03:01-DQB1*03:02) haplotypes and a lower risk with DR3-DQ2 (DQA1*05:01-DQB1*02:01 haplotypes (36). In addition to HLA class II susceptibility genes, several haplotypes confer a dominant protection, in particular the DRB1*15:01 (DR15)-DQA1*01:02-DQB1*06:02 (DQ6) haplotype through all stages of T1D (37). The association of different HLA alleles with T1D risk modulate the risk; combinations of HLA alleles can have additive or subtractive effects leading to an increase or a decrease in the T1D risk (35, 38–40). Heterozygous HLA-DQ2/8 genotypes confer the highest risk for T1D with OR that reach 40 (41). The HLA-DQ6/8 (DQA1*02:01-DQB1*06:02/DQA1*03:01-DQB1*03:02) genotype is protective (OR of 0.2) (41). The mechanism involved in HLA-DQ6 protection remains unknown. Recent publications demonstrate the complexity of HLA gene associations with the observation of autoreactive CD4^+^ T-cells restricted to HLA-DQ8 transdimers that form in HLA-DQ2/-DQ8 heterozygous individuals (42, 43). Children with heterozygous DR3-DQ2/DR4-DQ8 or homozygous DR4-DQ8 genotypes and a family history of T1D are four times more at risk to develop islet autoimmunity than children with the same genotypes but no T1D family history (32). In addition to HLA class II genes, several HLA class I alleles confer a susceptibility.

Non- HLA genes provide additional risk for T1D with lower odds ratios. In the TEDDY Cohort, 5806 subjects were genotyped for 176586 SNPs (33). Nine non-HLA genes including PTPN22, Ins, SH2B3, PxK5 were identified as increasing the susceptibility to T1D. Some of the identified genes function in β cells while others are involved in the regulation of immune responses or responses to viral infections (PTPN22, IFIH1). A VNTR located 5’of the *INS* gene (IDDM2) on chromosome 11p15.5 is the second most strongly associated locus with T1D risk (32). It regulates insulin expression in the pancreas and the thymus. Long and short VNTR are associated with high and low transcription of proinsulin mRNA, respectively, in thymus epithelial cells (15, 44). A low expression of insulin may favor thymic selection of CD4^+^ T-cells recognizing autoantigens (32). The protein Tyrosine phosphatase non receptor *PTPN22* gene on chromosome 1p13 encodes for lymphocyte specific tyrosine phosphatase (LYP) which downregulates TCR signaling, inhibiting T-cell activation (27, 45). A gain of function mutation in this gene may enhance T-cell suppression, leading to a decreased number and function of Treg cells and T1D susceptibility. *CTLA4* encodes for cytotoxic T-lymphocyte-associated protein 4 (CTLA 4) which acts as a costimulatory receptor on CD4^+^ T-cell and inhibits their activation by binding to B7. A decreased expression leads to increased T1D susceptibility (27, 46). Inducible T-cell COStimulator (ICOS, CD278) gene is expressed on activated CD4^+^ T-cells. It produces a stimulating T-cell activation signal and is involved in the control of regulatory CD4+ T-cells (47). The **A946T variant of the** Interferon induced with helicase C domain *IFIH1 (MDA5)* encodes an innate immune receptor that senses viral RNA, induces an interferon mediated virus resistance and **increases the risk for autoimmunity(** 48 **)**. Among other genes reported as susceptible genes, STAT 4, IL2, SUMO4, IL2RA have all been involved in T-cell regulation (46).

## Environmental Risk Factors

The lack of full concordance between homozygotic twins, the increasing incidence of T1D over the years, the change in T1D incidence among immigrants from a low-risk country to a high-risk country and the difference in risk between children of an affected father (7% risk) and of an affected mother (3% risk) underscore the role of environmental factors in T1D (2, 36). This explains the dilution of the frequency of high-risk HLA genes in the T1D population, such as the DR4-DQ8 or DR3-DQ2 genotypes in T1D children declined over the past 40 years (49, 50). Environmental factors are thought to play a role both in the triggering and in the progression of islet autoimmunity. In children with close genetic susceptibility, the incidence of overt T1D increased without an increase in the incidence of prediabetes (2, 51).

Many hypothetical models may contribute to explain how the environment triggers T1D. The hygiene hypothesis postulates that decreasing childhood infections linked to a better hygiene impairs the development of the immune system, limits the competition between self and non-self and disturbs T-cell regulation (52). The accelerator hypothesis – β cell stress hypothesis – postulates that insulin resistance secondary to accelerated growth or rapid weight gain triggers endoplasmic reticulum stress in β cells and possibly autoimmunity (53). The fertile field hypothesis suggests that infections would make the body more prone to develop autoimmunity by favoring responses to autoantigen. The “old friends hypothesis” implies that the lack of exposure to normal microbiota impairs the immune system regulation and maturation (54). Finally, the threshold hypothesis, which calculates and quantify the contribution of each genetic and environmental factor, suggests that an accumulation of events induces enough stimulation to overcome T-cell activation thresholds and trigger immune responses (55).

Many studies were conducted to study the influence of environmental factors on the development of islet autoimmunity and T1D in children. TEDDY has already evaluated candidate environmental triggers, including infections, probiotics, micronutrients, and microbiota (9). Potential factors influencing the risk of T1D have been identified (56). For many of these factors, the evidence is low and often controversial.

### Viruses

Epidemiological studies support the role of viruses in T1D. Enterovirus infections have been detected along T1D development and antiviral responses observed with autoantibody conversion in prospective studies (57). Upregulation of type I IFN genes has been reported in peripheral blood mononuclear cells of susceptible children prior to autoantibody conversion (58). Analyzing whole blood RNA transcriptomes brought the evidence of activation of innate immunity prior to seroconversion (59). Tissue samples from the Diabetes Virus Detection (DIVID) study showed that upregulation of IFN-stimulated genes correlated with insulitis (59). A viral infection could increase the risk of T1D directly through β cell cytolysis or indirectly by triggering diabetes immune activation. Infections could induce autoimmunity progressively rather than acutely, explaining the absence of viremia in children with rapid onset T1D in TEDDY (60). Many viruses are potentially involved: DNA viruses such as Epstein-Barr virus (EBV) and cytomegalovirus (CMV), RNA viruses, i.e., Rubella rotavirus and Enteroviruses (61). Enteroviruses were the most likely candidates for triggering T1D. Enterovirus RNA has been isolated from blood, stools, and pancreatic tissue of T1D patients (62, 63). Human pancreases showed enterovirus VP1 staining in almost all islets in 60% of recent-onset T1D patients while it was detected in few islets and only 6% of non-diabetic controls (64). Among Enterovirus B strains, studies focused on Coxsackie virus B (CVB) 1 to 6 and echoviruses. While mouse models have shown a direct relationship between viral infections and T1D, the evidence in humans is most often from epidemiological studies and does not establish a causative link. Many studies used serology to detect infections (65) although the detection of IgG antibodies does not identify a recent infection nor can it rule out an underlying silent chronic infection, in contrast with detection of viral shedding in stools, blood, or tissues by PCR or *in situ* hybridization. A positive association between Coxsackievirus B4 (CVB4) and T1D and Coxsackievirus B1 and the risk of islet autoimmunity has been reported using PCR-based detection methods (66, 67). The association between viral infections and T1D depended on whether the outcome was prediabetes or overt T1D. In TEDDY, EV-B increased by 4 times the risk of islet autoimmunity, but not T1D (68). However, CVB4 was the only serotype to be significantly associated with autoantibody conversion. The MIDIA study showed no association between viral shedding and the risk of islet autoimmunity (69). The DAISY study showed that detection of enterovirus in serum predicted the progression from autoimmunity to T1D (70). Adenovirus C was associated with a lower risk of islet autoimmunity in children aged 3 to 6 years (68). Screening for a viral infection is complex since variations might be seen due to intermittent shedding of the virus in stools. Detecting a viral infection have evolved from a sample every 3 months to sequential sampling to increase the likelihood of detecting transient viral RNA in stools.

The viral infection of the pancreas may have different consequences. CVB infects the pancreas, the islets and β cells although with different outcomes depending on serotypes. While some serotypes destroy β cells and are eliminated, others are less harmful and capable of low-grade proliferation while transfecting nearby cells through cellular vesicular trafficking mechanisms without lysing β cells (63). Beyond serotypes, the age at infection, the latency period from infection to seroconversion and the chronicity of the infection influence the autoimmune risk. While many enterovirus serotypes can associate with the autoimmune risk, in the DIPP case control study of 411 children, cases with islet autoimmunity showed high infection rates especially with Enterovirus-A prior to seroconversion (71). However, in the TEDDY cohort on 383 children, Enterovirus-B infection was associated with islet autoimmunity (68). Time lapse from infection to autoantibody conversion varied from months to years, suggesting a long-term effect of the virus (68). The prolonged infection determined by the duration of viral shedding significantly increased the risk of islet autoimmunity, possibly due to chronic islet inflammation. Other viruses have been associated with T1D. Recurrent respiratory viral infections at an early age was associated with higher risk of T1D development by the age of 8 (72). Congenital rubella induces T1D in 12 to 20% of children (73). The rubella virus can infect pancreatic β cells. It was observed that children with rubella-induced diabetes had a high frequency of HLA-DR3 and islet cell antibodies (73). Rotavirus and cytomegalovirus have also been implicated78, with controversial results (74, 75). A drop in T1D incidence has been observed following Rotavirus vaccination (76).

COVID-19 is a new potential trigger of T1D as activating innate immunity or increasing β cell stress (77). But data on the effect of COVID-19 on T1D incidence are conflicting (78). While some studies showed that COVID-19 exacerbate T1D and induce diabetic ketoacidosis in patients with new-onset T1D, other studies showed an increased incidence of T1D among patients with COVID-19 (79, 80). Centers for Disease Control and Prevention (CDC) report on January 7th, 2022, showed that COVID-19 infection increased the risk of T1D in patients below 18 years of age over 30 days following the infection (81). Nevertheless, whether Angiotensin-Converting Enzyme 2 (ACE2) is expressed on β cells or ductal cells is still discussed (82).

In addition to the triggering of T1D by infections, a protective effect of helminth infections has been reported (83).

### Early Life Diet

Food introduction in neonate and early diet have been reported to impact T1D development. Diet may contribute to T1D by introducing cross-reactive antigens or by interfering with the gut microbiota. A systematic review and metanalysis to study the effect of dietary factors on the risk of islet autoimmunity and T1D showed a moderate to high level of certainty that a longer breastfeeding and later gluten and cow’s milk introductions reduced the risk of T1D (84). So far, randomized clinical trials failed to show a beneficial effect of dietary intervention on the risk of T1D (84). Many studies have shown an association with T1D but the causality is still to be proven (55).

Different dietary factors have been related to the risk of T1D. The role of breastfeeding on the risk of T1D remains unclear and the consequences of initiating or prolonging breastfeeding is still controversial (85–87). While there were no associations between breastfeeding and autoimmunity in the DAISY (88) and the MIDIA studies (86), an association was identified in two Scandinavian cohorts (87). Breast milk has an impact on gut microbiota. The predominant bacteria are the butyrate producing Firmicutes and actinobacteria (89). Introducing breast milk at an early stage increased the infants’ gut colonization by Bifidobacterium species in the TEDDY cohort (90). The duration of breastfeeding had no significant effect. A shift in the gut microbiota was observed when interrupting breastfeeding but the effect of breastfeeding on gut microbiota was sustained. At the long run, it maintained a healthier gut microbiota. Introducing bovine milk before 3 months of age influenced T1D development. Cow’s milk intake was hypothesized to increase autoantibody conversion and is currently being studied in the Trial to Reduce IDDM in the Genetically at Risk (TRIGR) study (91). Studies have shown that in children, the greater the intake of cow’s milk products, the higher the risk of diabetes autoimmunity. A cross reactivity between alpha casein and insulin has been proposed (92).

Beyond breast feeding and introduction of bovine milk, both early and late solid food introduction were associated with T1D development in BABYDIET (93). However, the risk of T1D didn’t change regardless of the timing of gluten introduction. Gluten has long been considered as a risk factor for T1D due to the association of T1D with coeliac disease in 10% of T1D patients. In most cases, T1D precedes celiac disease. Gluten free diet decreased the incidence of T1D in NOD mice (94). The effect was more pronounced when the exposure to gluten is absent during fetal and neonatal development in NOD mice (94). These observations were also made in the BioBreeding (BB) rat (95). In humans, the effect of early childhood intake of gluten and fibers on T1D is controversial. DAISY, and TEDDY studies showed no association between early gluten and fiber intake and the risk of autoantibody conversion nor the risk of progression to overt diabetes (96, 97). In BABYDIET, no difference was seen between early versus late gluten introduction in children (98). The DIPP study showed that early and higher gluten and fiber intake increased the risk of autoantibody conversion (99). Another cohort showed an association between early gluten intake and the risk of T1D (100) and one study showed that to prevent the increase in T1D incidence, cereals should not be introduced in 4 to 6 years old children. An early introduction increased the risk of T1D by 4 (101). Undigested gliadin can irritate the intestinal mucosa and, in some people, induce mucosal inflammation which increases the gut permeability and allow the migration of immune cells, cytokines and gliadin antigen to adjacent organs. Gliadin from the bloodstream can deposit in the islets, activate gliadin autoreactive T-cells and induce insulitis (102, 103). However, in some experiments increased gluten intake in NOD mice prevented T1D (94).

Finally, higher carbohydrate and sugar intake were found to increase the risk of progression from islet autoimmunity to T1D (104). Studies have suggested that sugar and carbohydrate intake are significantly associated with the risk of T1D with a low level of evidence.

### Microbiota

There is growing evidence of the contribution of the gut microbiota to T1D pathogenesis. In animal models, the risk of developing autoimmune diabetes was related to the gut flora. While lactobacillus and bifidobacterium were associated with resistance to diabetes, bacteroides were associated to diabetes in diabetes-prone BB rats (105). NOD mice that received antibiotics and germ-free NOD mice showed accelerated diabetes (106–108). Antibiotics were protective in BB rats, but they accelerated diabetes in the mouse. Factors that shape the microbiota are mostly environmental factors (109). The diet provides the substrate promoting the survival and proliferation of some strains at the expenses of others (110). Bacterial exposure during the early life can also modulate the microbiota. Genetic factors might favor a dysbiosis immune tolerance failure and promote auto-immunity. Some studies showed an association between a T1D susceptibility genes and altered gut microbiota (111). Any modification in the composition of the microbiome can impact the mucosal integrity and immune tolerance. Differences are observed between the gut microbiota of diabetic and healthy individuals (112). The microbiota in T1D patients is less diverse and rich in harmful organisms. Gut microbiome influences the risk of T1D by increasing permeability, by modulating the gut immune system or by molecular mimicry.

Microbiota is affected by dietary habits which render the study of its effect on human T1D difficult. Autoantibody conversion was influenced in children by the diversity of the microbiota, the type of bacterial genera and short chain fatty acid (SCFA) production. In the DIPP study, differences were seen in the microbiome of seroconverted children as compared to controls (113). A decline in Firmicutes and an increase in Bacteroidetes was observed in patients with islet autoimmunity. Bacteria that are known to produce butyrate SCFA were predominant in controls. Butyrate could prevent T1D by reducing gut permeability, by limiting bacterial translocation, by inducing immune tolerance by regulatory T-cell (Treg) expansion and by stimulating the secretion of anti-inflammatory cytokines (114). TRIGR and FINDIA cohorts found that the loss of diversity was associated with autoantibody conversion and that lactate and butyrate were negatively correlated to the number of autoantibodies (115). In the DIABimmune study on 33 genetically predisposed children, a shift in the microbiota was observed in autoantibody-positive children who progressed to overt diabetes (116). In TEDDY, the analysis of metagenomes and 16sRNA genes in the stools of children from 3 European countries and 3 American states as of 3 months until autoantibody conversion or T1D development showed a higher expansion of streptococcus species at the expense of Lactobacillus rhamnosus and Bifidobacterium dentium in autoantibody-positive individuals (90). However, T1D patients had more *Bifidobacterium pseudocatenulatum, Roseburia hominis, and Alistipes shahii* as compared to controls. Microbiota in diabetic cases was associated with a decreased production of acetate, butyrate, and propionate SCFA. No consistent association was found between antibiotics intake and the risk of T1D in humans.

### Other Factors

Less common factors were also incriminated in T1D development.

#### Antenatal and Perinatal Factors

No major associations were seen between T1D or islet autoimmunity and maternal age, maternal obesity, delivery route, and vitamin D levels in pregnancy (117) and at birth (118). But maternal infection during pregnancy has been suggested as a risk factor for T1D. However, a consistent association between the two variables was never established due to heterogeneous study designs. A meta-analysis revealed an association between virus infections in pregnant women and subsequent development of islet autoimmunity or T1D in their children (119). The Danish National Birth Cohort study showed that the association between gluten rich diet during pregnancy and the risk of developing T1D in the child is borderline (120). By contrast, MoBa study failed to show any association (100).

#### Infancy and Childhood Factors

Evidence of associations of birth weight, obesity, and body mass index (BMI) with T1D is conflicting. The assessment of the causality is suggested by the association of obesity related genes and T1D risk. A meta-analysis showed a positive association between childhood obesity or BMI and T1D, with an estimated relative risk of 1.2 (121). However, no associations with seroconversion or the rate of progression from islet autoimmunity to T1D were detected in longitudinal cohorts on children born with high susceptibility except with high growth velocity (122).

Although adults with new onset T1D had a lower vitamin D level than the healthy controls, Vitamin-D (25(OH)D) level did not correlate with islet autoimmunity or T1D development in children (123). These findings contradicted the mild protective effect of low levels of 25(OH)D seen in the 2018 TEDDY study (124). A systematic review and metanalysis assessing the association between six genes implicated in Vitamin D pathway and the development of T1D found no genetic associations (124).

Blood levels of omega-3 fatty acids in children prior to T1D diagnosis were also linked to T1D development. A higher level of DocosaHexaenoic Acid (DHA) in 3 months old infants was associated with a decreased risk of islet autoimmunity (125). Similarly, in the DAISY study, a higher omega-3 intake protected children from seroconversion but not from overt disease (126).

A systematic review assessing the link between vaccination and childhood onset of T1D failed to show any significant association (127). On the contrary, a possible protective effect of the rotavirus vaccination against T1D development was suggested in Australia (76) and USA (128). These observations were not replicated after 12 years follow up in a Finnish randomized clinical trial (129).

A recurrent statement links psychological stress to the induction of T1D. Stress increases stress hormone concentrations, contributing to resistance to insulin. Retrospective studies showed a positive correlation between stress and the risk of T1D. However, the perceived stress in these studies might have been related to the diagnosis of T1D rather than other stressful life events. A psychological stress assessment of 58 T1D children from the prospective ABIS studies showed that a childhood exposure to a serious life event increases the risk of T1D with an estimated HR of 3 (130).

#### Adulthood Factors: Immune Checkpoint Inhibitors

The incidence of T1D with anti-PD1, with anti-PDL-1, with a combination of anti-PD1/anti-PDL1 and with anti-CTLA-4 is respectively 1.18%, 0.73% and 2.6% (131). Associated factors explaining why some people develop T1D while others do not, remain to be identified. ICI play a role in maintaining immune tolerance to β cell autoantigens. In murine models, treating NOD mice with anti-PD1 or anti-PDL1 accelerated T1D at any age. In contrast, anti-CTLA-4 administration induced diabetes only when injected in neonates (132). Also, invalidation of PD-1 or PDL-1 in NOD mice accelerated the development of T1D, whereas it did not induce T1D in other mouse strains (133). This shows that other players may contribute to the risk of developing T1D.

## Mechanisms Behind Autoimmunity

The crosstalk between β cells and the immune cells, between innate and adaptive immunity and between both the immune system or β cells and the environment are central in the development of T1D. T1D susceptibility genes that directly drive immune interactions with environmental factors have been identified. β cells are also central in interactions with the environment beyond the role of nutritional factors.

### Role of β Cells

β cells express virus receptors for coxsackievirus and adenovirus receptors (CAR). Among the five CAR isoforms, human β cells express the CAR- simian immunodeficiency virus (CAR-SIV) isoform that carries the transmembrane domain that allows virus entry in cells (134). The expression of CAR is mainly in the insulin secretory granules. Viral infection of β cells allows its replication and leads to β cell destruction. Exposure of human CBV-1 infected islets to CXCL-10 increases CAR expression in β cells (135).

In addition to direct destruction of β cells, viral islet infections induce indirect lesions that relate with endoplasmic reticulum (ER) stress and oxidative stress (4). When stressed, β cells adapt by upregulating the unfolded protein response (UPR) and decreasing insulin synthesis and secretion. Stressed β cells upregulate surface expression of HLA class I molecules, initiate degradation of proteins in the ER, promote apoptosis, autophagy, and necroptosis to restore homeostasis. The release of danger signals such as chemokines attracts immune cells to relocate into the islet and leads to islet inflammation (136, 137). Subsequent changes in β cells induces their senescence and further increases intra-islet immune cells infiltration. Studying the molecular footprint of stressed β cells allows to characterize the stressor (10). The link between stressed β cell and the induction of T1D is evident under the action of toxins such as alloxan or streptozotocin (138). By entering in β cells through GLUT2, alloxan generates reactive oxygen species (ROS) in the presence of glutathione, which eventually induces β cell death (139). Streptozotocin (STZ) induces DNA damage and ROS generation along with impairment of insulin secretion, leads to insulitis in some mouse strains and to β cell apoptosis and death (140). The role of ER and oxidative stress in T1D was also suggested in studies on human pancreatic islets. Genes involved in ER stress were upregulated in the diabetic islets, with evidence of UPR stimulation (141, 142). In mouse models, inhibiting the ER stress with tauroursodeoxycholic acid delayed β cell destruction (143).

An additional ER stress factor is the low β cell mass, which increases the burden on β cells to maintain normoglycemia, especially in circumstances when insulin requirement increases. It creates a vicious circle in prediabetes once the β cell mass starts to decrease under the development of insulitis. Hyperinsulinemia was detected in patients’ blood prior to T1D development (144). Aggravating situations that lead to higher β cell pressure relates to diet, accelerated weight gain, growth acceleration at puberty and insulin resistance in the last trimester of pregnancy, all situations that often reveal T1D (145). However, resting β cells by insulin injections was immunogenic and failed to prevent T1D in the Diabetes Prevention Program Trial (146).

Another predisposing factor of β cell ER stress is a proinflammatory microenvironment caused by a viral infection or by a leaky gut secondary to microbiome changes. Proinflammatory environment activate the STAT1, IRF1 and NFκB pathways and upregulate the expression of HLA class I on β cells (147–149). β cell exposure to Il-1B, TNF alpha and IFN gamma induces an oxidative stress (150). β cells are characterized by low antioxidant capacities. Oxidative stress leads to misfolding and accumulation of proteins in ER, exacerbating the ER stress (151). Oxidative stress upregulates chemokine receptor expression, activates NFκB pathway and stimulates cytokine secretion which further attracts immune cells to the pancreas, creating a vicious cycle of inflammatory reactions and promoting islet autoimmunity. T1D patients exhibit a higher level of oxidative stress markers in their serum when compared to controls (152). Chronic inflammation induces further β cell lesions which could explain the risk of islet autoimmunity in cases of chronic viral shedding. Mitochondrial dysfunction also increases ROS accumulation, which increases the risk of T1D (153).

### Immune Mediated β Cell Destruction

#### Innate Immunity

As previously shown, activation of innate immune responses induces β cell damage through oxidative stress, ER stress, cytokine toxicity, phagocytosis. Even an adjacent injury to β cells, in particular in endothelial cells, can induce β cell damage through a bystander effect. Viral infections activate innate immunity after phagocytosis of infected cells or cellular debris. The innate immune response is initiated once pattern recognition receptors (RIG-1 like receptors, TLR) whether in the cytoplasm or on cell membranes, are stimulated by viral particles or β cell debris (ssRNA, DsRNA and CpG containing DNA) leading to INFα production by β cells and driving inflammation and antigen presentation (154). Transcriptomic studies after *in vitro* stimulation of β cells from recent-onset and long-standing T1D showed early activation of pattern recognition receptors (PRR), upregulation of major histocompatibility complex (MHC) class I expression. In a later phase, T-cell activation and recruitment profiles and upregulation of cytokines and immune check point molecules are observed (10). The recognition of host nucleic acids by TLR 3, 8 or 9, retinoic acid inducible gene (RIG-1) or melanoma differentiation-associated protein 5 (MDA-5) leads to transcriptional expression of inflammatory mediators to eliminate pathogens, but also activate an autoimmune response, as a side effect. Polymorphisms in *TLR3* and *MDA5* genes have been associated with the risk of T1D (34). The role of TLR3 was shown by inducing autoimmune diabetes by polyI:C in animal models, in particular in TLR-9^-/-^ or TLR-3^-/-^ transgenic mice expressing the co-stimulation molecule in the pancreas,. However, administering antibiotics with polyI:C induced diabetes only in TLR-9^-/-^ mice whereas TLR-3^-/-^ mice were protected. Activation through TLR-3 was independent of microbiota, as opposed to activation through TLR-9 (155). Data from GWAS showed that a partial loss of function of MDA5 prevents T1D (156). The gain of function of MDA5 in C57B6 *Ifih1* ^R/R^ mice increases T1D risk (48).

Stimulated TLR interacting with NOX2 (NADPH oxidase 2) or NOX4 (NADPH oxidase 4) activates NFκB pathway, produce superoxide and potentiate the inflammatory responses (157, 158). This was seen when TLR-3 was stimulated which shows the importance of TLR-3/NOX2 interaction in producing an anti-viral response (159). Viral infection with DNA parvovirus or Kilham Rat virus triggers autoimmune diabetes in 30% of diabetes-resistant BB rat. Co-administration of polyI:C can increase the incidence to almost 100% (160).

IFNα, a modulator of inflammatory and ER stress responses, is a key player in initiating an innate immune response in the early stage of T1D (161, 162). Infections of β cells and any tissue stress or damage lead to the production of IFN α, thereby promoting a diabetogenic environment. This activates the expression of adhesion and co-stimulation molecules, HLA class I molecules, cytokines, and chemokines, and recalls antigen presenting cells, NK cells and T-cells to the injured site. Along the same line, several IFNα signaling and antiviral immune response genes that have been associated to T1D in prediabetic children (163). Studies of pancreatic tissues obtained from the network for pancreatic organ donors with diabetes (nPOD) database has detected upregulation of IFNα, of IFNα-related genes and overexpression of HLA class I molecules. IFNα secreting plasmacytoid dendritic cells were isolated from the blood of T1D patients (164, 165). Finnish (DIPP study) (58) and German (BABYDIET study) cohorts (59) revealed that IFNα signature is temporally increased in susceptible children prior to the detection of autoantibodies, but not in established T1D. A study comparing gene expression profiles of circulating blood cells from children at the onset of T1D, autoantibody-positive first-degree relatives of T1D children and healthy controls identified 107 genes that were differentially expressed. Among these, a major gene cluster was regulated by IFNα (166). IFNα links innate immune responses to the adaptive immune response by promoting antigen presentation and activation of T-cells. IFNα activates ER stress and apoptosis, increases alternative splicing, and inhibits β cell regeneration. IFNα also induces mitochondrial dysfunction in β cells, impairs insulin secretion and promotes β cell apoptosis (167). The latter will increase IFNα secretion and perpetuate the immune response.

Macrophages are seen in the islets of patients with recent onset T1D. They are among the first cells to be identified in the pancreas in animal models of T1D. T1D is prevented in NOD mice that have been depleted of macrophages (168). In the human, gene variants that control macrophage activation show an association with T1D. Macrophage polarization towards a proinflammatory M1 phenotype is observed following pathogen associated molecular patterns (PAMPS) binding to TLRs along with NFκB activation in pro-inflammatory environments. Activated macrophages secrete free radicals, cytokines, and chemokines to eliminate pathogens, initiating a chronic islet inflammatory state that ultimately triggers autoimmune activation on genetically susceptible genetic backgrounds. A defect in phagocytosis of apoptotic β cells can further favor autoimmune activation. Macrophages may also contribute to T1D pathogenesis due to an aberrant antigen presentation secondary to inefficient protein processing (149, 150, 169). They can deviate antigen presentation towards activation of autoreactive T-cells (169).

#### Adaptive Immunity

The innate immune response seen in the first stages of autoimmune development triggers the activation of autoreactive T and B cells and their recruitment to the pancreas. As gene variants favor the development of an islet proinflammatory response, other variants favor the failure of both central and peripheral immune tolerance to β cell autoantigens. In addition to conventional autoantigens and the proinflammatory islet context, neoepitopes that have been described may contribute to immune tolerance failure to β cells. Moreover, environmental factors such as vitamin D deficiency may contribute to autoreactive T-cell activation by modifying T-cell regulation (170).

Seemingly, microbiota changes in T1D have been related to the adaptive immune response to β cells. SCFA, the production of which directly depends on the microbiota composition, has shown a protective effect in NOD mice along with increased Treg frequencies and decreased autoreactive T-cell frequencies. Low SCFA levels have been observed in human T1D in relation with microbiota changes. It has further been shown that bacterial mimics of β cell autoantigens are recognized by T cells. SCFA also stimulate the production of antimicrobial peptides from β cells and activate innate immune cells which reverts the proinflammatory profile of antigen presenting cells (171). SCFA and the anti-microbial peptides (cathelicidin-related antimicrobial peptide (CRAMP)) protected NOD mice from autoimmune diabetes (171). However, restoring the integrity of gut mucosa in clinical trials using butyrate failed to induce changes in monocyte subsets and *in vitro* cytokine production (172). Microbiota plays a role in the modulation and the maturation of the immune system (173). Germ free mice require colonization with commensal bacteria to develop a Th17 and Treg cell response in the gut (174). Beyond its effect on the immune system, microbial dysbiosis induces a proinflammatory environment in the gut, increasing gut permeability and allowing the translocation of microbial and inflammatory side products as well as antigens to adjacent organs such as the pancreas (175).

The MHC affects central tolerance by shaping the T-cell repertoire in the thymus. Islet inflammation creates the conditions for breaking tolerance of autoreactive T-cells. Environmental factors also likely contribute to break peripheral tolerance by producing diabetogenic neoantigens through mutations, frameshifts, alternative mRNA splicing and post-translational modifications (4). Combined effects of physiological ER stress and the various stresses induced by environmental factors may contribute to antigen spread and hasten disease onset. The ER stress promotes the production of aberrant translation products such as defective ribosomal products (DRiPs), modulates the translation initiation process and affects the degradation of insulin byproducts. ER stress activates post-translational modification (PTM) that contribute to the formation of neoepitopes such as trans-glutaminated chromogranin A or palmitoylated GAD, capable of activating specific CD4^+^ T-cells (176, 177). Hyperglycemia perpetuates ER stress, thus leading to more PTM and more neoepitopes generation (178).

Environmental factors can finally activate diabetogenic T-cells through molecular mimicry based on structural similarities between islet autoantigens and pathogens as proposed in the case of cytomegalovirus (CMV), thereby initiating a cross-reactive autoimmune response. Based on a higher incidence of infections with Mycobacterium avium paratuberculosis in Type 1 diabetic patients in Sardinia, a study has shown a cross-reactivity with ZnT8 which could explain a high T1D incidence (179). Another study showed cross-reactivities between hGAD-65 and the gut bacterial GAD with an identified bacterial GAD peptide overlapping with a hGAD-65 specific TCR epitope (180). But more studies will be required to demonstrate the role of molecular mimicry. Induction of T1D is the resultant of the alignment of β cell dysfunction and a pro-inflammatory microenvironment. Auto-immune diabetes was successfully induced in a BALB/C DEREG mouse model using the double-hit strategy starting with a low dose STZ injections to release autoantigens followed by Treg cell depletion (181).

## Conclusion

Although many environmental factors have been proposed to trigger or modulate the development of T1D in individual at risk of developing the disease, no unique environmental factor has so far been involved. It makes T1D a highly multigenic disease that exemplifies an unbalanced interaction between a highly multigenic predisposition background and a rapidly changing environment. In genetically predisposed individuals, T1D may be triggered because of the failure of intrinsic tolerance mechanisms and/or environmental triggers. The best predictor for T1D development is seroconversion when two or more autoantibodies are detected. Studies aimed to refine this tool by using longitudinal sampling of islet antibodies. This approach identified three distinct trajectories that predict the progression from latent to overt T1D based on clinical and serological features (182). Genetic factors also play a role in predicting T1D development (32, 183). The prediction power of a model is improved by using combined putative HLA and non-HLA genes. Recently, an updated genetic risk score (GRS2) was published (184). It captured information on 67 SNPs in non-HLA genes and 18 HLA DR-DQ combinations in HLA genes and outperformed the other genetic risk scores in screening for T1D in newborns and in classifying adult-onset diabetes. However, genetic predisposition is not the only determinant of T1D. Combining GRS2 to longitudinal autoantibody measurements and family history doubled the screening efficiency in newborns as compared to serology alone (185). So far, a T1D screening tool combining genetic and environmental factors was not successfully developed. If it existed, this tool would estimate T1D risk in children and adults based on the situation of each individual. A heterogeneity is seen in the clinical presentation of patients with T1D. The age at onset, the kinetics of β cell destruction, the time from initial autoantibody detection to development of hyperglycemia, the number of β cell autoantigens against which autoantibodies are detected, the weight of the familial history of diabetes, and the HLA genetic background differ from one patient to another. Environmental factors induce epigenetic modifications such as DNA methylation, histone modification and microRNA production that are possibly involved in the development of islet autoimmunity (186–189). Many SNPs identified by GWAS studies map in non-coding regions, especially in enhancers are likely to control gene expression. DNA methylation patterns have been identified in T1D, as well as histone modification profiles (190). The methylation status of genes that are known to contribute to the risk of T1D have been identified. Evaluating their impact on islet autoimmunity development will be crucial. So far, their link with identified environmental factors is still missing. Large cohort studies combining deep clinical phenotyping and record of large data sets for environmental factors to cutting edge multi-omics including (epi)genomics, proteomics metabolomics, and spatial transcriptomics at cellular resolution should enable a more precise delineation of molecular mechanisms of T1D leading to reclassification (or clustering) of T1D endotypes based on primary molecular pathogenesis. As for mechanisms involved in the interaction between the environment and the gene variants identified in T1D susceptibility, it will be crucial to explore all the possible molecular windows that allow screening the effect of environmental factors on living organisms. Beyond the microbiota, the study of epigenetic modifications such as DNA methylation, histone modification and microRNA production opens new windows to study the impact of environmental factors.

## Author Contributions

All authors contributed to the article and approved the submitted version. PH designed, did the literature review, and wrote the article. SL designed, reviewed, and edited the review. CB designed, reviewed, and edited the review.

## Conflict of Interest

The authors declare that the research was conducted in the absence of any commercial or financial relationships that could be construed as a potential conflict of interest.

## Publisher’s Note

All claims expressed in this article are solely those of the authors and do not necessarily represent those of their affiliated organizations, or those of the publisher, the editors and the reviewers. Any product that may be evaluated in this article, or claim that may be made by its manufacturer, is not guaranteed or endorsed by the publisher.
